# Intelligent prediction of thyroid cancer in China based on GBD data and hospital electronic medical records: disease burden analysis combined with multiple machine learning models

**DOI:** 10.3389/fendo.2025.1644396

**Published:** 2025-08-20

**Authors:** Lina Yang, Shixia Zhang, Xinguo Wang, Jianjun Yang, Mengya Chen

**Affiliations:** ^1^ Development Department of the Wisdom Hospital, Shandong Provincial Third Hospital, Jinan, China; ^2^ Department of General Practice, Shandong Provincial Third Hospital, Jinan, China; ^3^ Department of Medical Records, Shandong Provincial Third Hospital, Jinan, China

**Keywords:** thyroid cancer, disease burden, body mass index, EMR, Mendelian randomization, machine learning

## Abstract

This study aims to conduct an in-depth analysis of the disease burden pattern and future trends of thyroid cancer in China, and constructed an intelligent prediction model in combination with hospital electronic medical record data. It comprehensively reveals the disease burden trend of thyroid cancer in China, predicts the mortality rate of thyroid cancer in China, and emphasizes the causal role of high BMI as an important controllable risk factor. And provided a high-precision prediction model for benign and malignant thyroid cancer. The results show that the prevalence of thyroid cancer in China has shown a significant upward trend from 1990 to 2021, especially among women, and the peak age of onset has shifted later. The mortality rate of men is on the rise, while that of women is on the decline. The risk of thyroid cancer mortality caused by high BMI significantly increases during this period, and MR analysis confirms that high BMI increases the risk of thyroid cancer. The ARIMA model predicts that the prevalence of thyroid cancer in China will continue to increase in the next ten years, while the mortality rate will remain relatively stable. Among the machine learning models, XGBoost achieved the highest predictive accuracy and identified BMI as the most influential clinical feature in distinguishing between benign and malignant thyroid tumors. This study provides a solid scientific basis for the development of more accurate and effective strategies for the prevention, early diagnosis, and management of thyroid cancer in China and even globally, and provides a feasible path for the use of artificial intelligence assisted diagnosis in clinical practice.

## 1 Introduction

Thyroid cancer is one of the most common endocrine malignancies and ranks prominently among head and neck cancers worldwide, accounting for about 3% to 4% of all cancer cases (1, 2). In the past four decades, the prevalence of thyroid cancer worldwide has shown a significant upward trend in many countries (3).

The thyroid gland is a butterfly-shaped gland located in the front of the neck. Its main function is to secrete thyroid hormones (4), which regulate almost all physiological activities such as metabolism, energy balance, heart rate, body temperature, and even brain development. Therefore, maintaining normal thyroid hormone levels is essential to maintaining good health (5). However, when this critical gland becomes cancerous, the effects are profound and complex. As the most common malignant tumor of the endocrine system, thyroid cancer has a profound impact on the patient’s quality of life and long-term prognosis (6). These impacts are not limited to the physiological level, but also include psychological and social dimensions, making the management of thyroid cancer a complex process that requires comprehensive consideration (7).

To fully understand and address the increasing disease burden of thyroid cancer, the GBD data provides a valuable and publicly accessible comprehensive database, covering detailed data on 354 diseases and injuries worldwide (8, 9). The GBD data can effectively identify the latest trends and changes in thyroid cancer. Existing studies have extensively explored the overall global trends of thyroid cancer (10), and conducted specific analyses on the epidemiology of thyroid cancer within China (11–13). In addition, some comparative studies have contrasted the trends of thyroid cancer in different sociodemographic index (SDI) regions, between China and the United States, within the European Union, and among certain countries (14–17). However, at present, there are still obvious deficiencies in the comprehensive comparative analysis of the trend of thyroid cancer in China. Combining GBD macro-level data with micro-level electronic medical records offers a comprehensive view that bridges national trends and clinical realities. This kind of comparative analysis is crucial for China to identify its room for improvement in disease prevention and control and draw on the successful experiences of other countries.

This study uses GBD data to analyze and compare thyroid cancer patterns in China and predict future trends. The research analyzed key indicators such as the prevalence rate and mortality rate, and deeply explored the influence of factors such as gender and age on the disease burden. Furthermore, given that a high body mass index (BMI) is regarded as an important risk factor for thyroid cancer (18), this study will further explore the risk of attributable death and its causal relationship with thyroid cancer. Combined with the outpatient data of hospitals, the machine learning model is utilized to predict the future prevalence trend of thyroid cancer, with the expectation of providing empirical basis for formulating more precise prevention, control and treatment strategies for thyroid cancer in China and even globally.

## 2 Materials and methods

### 2.1 Materials

#### 2.1.1 Global burden Of disease data

This study will utilize the publicly available data from the GBD study in 2021 to obtain key epidemiological indicators such as the prevalence and mortality of thyroid cancer in China and globally from 1990 to 2021. Thyroid cancer is divided into various types, including papillary carcinoma, follicular carcinoma, poorly differentiated carcinoma, undifferentiated carcinoma, and medullary carcinoma. The diagnostic codes for thyroid cancer in this study include C73-C73.9, D09.3, D09.8, D34-D34.9, D44.0, and Z85.850 in ICD-10.The GBD data is released by the Institute for Health Metrics and Evaluation (IHME), providing a comprehensive assessment of the burden of disease at the global, regional and national levels, ensuring the authority and comparability of the data (19). All GBD data through its official database (Global Burden of diseases Study 2021, http://www.healthdata.org/gbd).

#### 2.1.2 Electronic medical record data

This study will collect the clinical data of patients with thyroid cancer from the Electronic Medical Records (EMR) system of our hospital. To ensure the consistency of data time, the time range ends with thyroid cancer patients who visited before 2021 (including 2021).The collected clinical indicators include the basic information of the patients (such as gender and age), physical examination data (such as BMI), and biochemical indicators related to thyroid function (such as TG, TP0AB, TGAB, TSH, FT4, FT3), as well as the final benign and malignant diagnosis results. These data are crucial for building precise machine learning prediction models. To ensure patient privacy and data security, all electronic medical record data will undergo strict anonymization and de-identification processing before analysis. To ensure data security, generate a unique ID for each patient that does not contain any personal information, and use this ID instead of the real identity in the analysis.

### 2.2 Methods

#### 2.2.1 The autoregressive integrated moving average model

The Autoregressive Integrated Moving Average (ARIMA) model is a widely used statistical method for analyzing and forecasting time series data. It works by analyzing the autocorrelation and non-stationarity present in the data. The core of the ARIMA model lies in its three components: the Autoregressive (AR) part uses a linear combination of past values to predict the current value; the Integrated (I) part addresses non-stationarity by differencing the data until it becomes stationary; and the Moving Average (MA) part uses a linear combination of past forecast errors to improve predictions.

An ARIMA model is typically denoted as ARIMA(p, d, q), where p is the order of the autoregressive component, d is the order of differencing, and q is the order of the moving average component. Building an ARIMA model involves several key steps: checking for stationarity, selecting appropriate model orders using Autocorrelation and Partial Autocorrelation Functions, estimating parameters, performing diagnostics, and generating forecasts. ARIMA is well-suited for time series data with strong autocorrelation and sees broad use in fields such as economics, finance, and meteorology. However, it assumes a linear relationship in the data and may not perform well for complex nonlinear patterns (20, 21). In this study, the ARIMA model was used based on historical GBD data to predict long-term trends in future prevalence and mortality rates, provided important evidence for public health decision-making.

The ARIMA model analysis was conducted in the R language environment, mainly utilizing the auto. arima() function in the forecast package. Before applying the ARIMA model, we first conducted a stationarity test on the original time series data. According to the test results, in order to eliminate the non stationarity of the sequence, we performed first-order differencing (d=1) on both the incidence and mortality data. The differencing operation ensured that the mean and variance of the sequence remained constant over time, meeting the requirements of ARIMA model for stationarity.

The auto. arima() function evaluates the goodness of fit of models with different combinations of p, d, and q based on the Akaike Information Criterion (AIC), and selects the optimal model. For the prevalence rate, the optimal model determined is ARIMA (1,1,1), with an AIC value of -140; For mortality rate, the optimal model determined is ARIMA (0,1,1), with an AIC value of -19.88. After the model is constructed, it is validated by checking the autocorrelation function (ACF) of the residual sequence. In addition, we also checked the normality distribution of the residuals to ensure the validity of the model assumptions.

#### 2.2.2 Mendelian randomization analysis

Mendelian randomization (MR) is a method used to infer causal relationships between exposure factors and disease outcomes (22). We employed it to analyze the association between body mass index (BMI) and thyroid cancer. The core idea is that if a genetic variation is strongly correlated with the exposure factor, independent of confounding factors, and affects the disease outcome through the exposure factor, then the association between this genetic variation and the disease outcome can be used to infer the causal relationship between the exposure factor and the disease outcome (23).

#### 2.2.3 Analysis machine learning prediction model

This study constructed and trained six machine learning classification models, achieving the prediction of benign and malignant thyroid cancer. XGBoost is a kind of efficient, flexible and scalable gradient boosting decision tree algorithm, which performs well in various prediction tasks (24, 25). RandomForest improves the prediction accuracy and robustness by constructing multiple decision trees and taking their average results (26). DecisionTree represents decision rules through a tree-like structure (27). Gaussian Naive Bayes (GNB) is a probabilistic classifier based on Bayes’ theorem, assuming that features are independent of each other and follow a Gaussian distribution (28). Multi-layer Perceptron (MLP) is a kind of feedforward artificial neural network that contains at least one hidden layer and is capable of learning complex nonlinear relationships (29). The Support Vector Machine (SVM) separates data points of different categories by finding the optimal hyperplane (30, 31).

## 3 Results

### 3.1 The prevalence rate of thyroid cancer in China from 1990 to 2021

**Figure 1:**
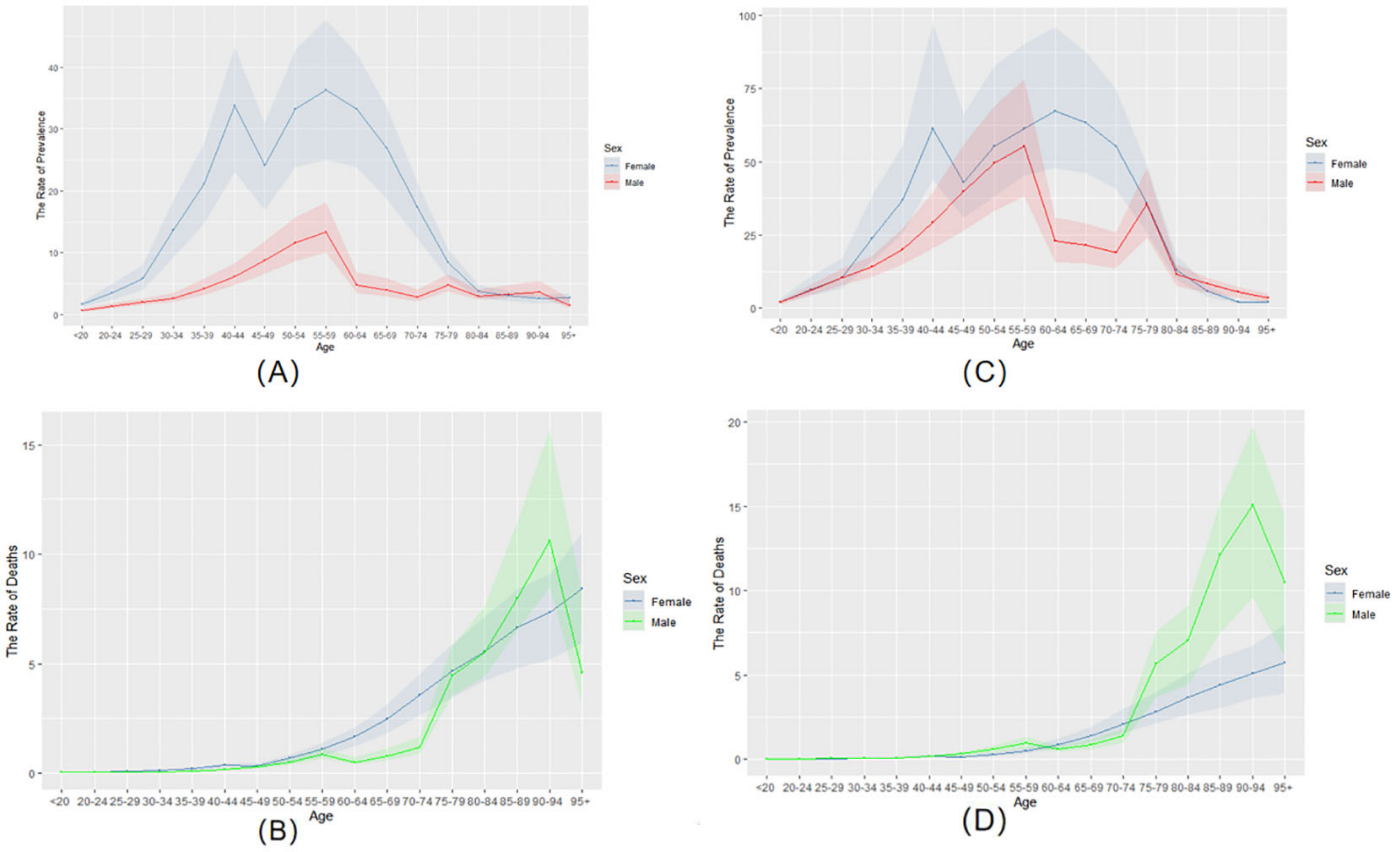
The prevalence and mortality rates of thyroid cancer in China from 1990 to 2021. **(A)** the rate of prevalence in 1990. **(B)** the rate of mortality in 1990. **(C)** the rate of prevalence in 2021. **(D)** the rate of mortality in 2021.

**Figures 1A, C** analyze the prevalence of thyroid cancer among women and men in China from 1990 to 2021. In 1990 (**Figure 1A**), the crude prevalence rate of thyroid cancer in China increased with age, and the prevalence rate peaked at the age of 55-59. However, in 2021 (**Figure 1C**), the peak prevalence rate for women shifted to the age of 60-64, while that for men remained at 55-59, showing a significant decline at the age of 60-64. Since 1990, the overall prevalence rate has shown a marked increase. For women, the prevalence rate is higher than that of men in most age groups. Moreover, the development trend of the disease among women is largely consistent, with a decline occurring between the ages of 45 and 49.

Between 1990 and 2021, cases per 100,000 people rose from 0.87 (95%UI 0.69, 1.0) to 3.88 (95%UI 3.12, 4.88). Among males, the number of affected cases increased from 0.19 (95%UI 0.15, 0.26) to 1.55 (95%UI 1.11, 2.08). The number of female patients increased from 0.68 (95%UI 0.49, 0.84) to 2.33 (95%UI 1.69, 3.46).

The ASPR of the entire population increased from 8.1 (6.41, 9.66) in 1990 to 20.01 (16.14, 25.23). The proportion of males increased from 3.52 (2.77, 4.73) to 15.87 (11.35, 20.96). The ASPR of females increased from 13 (9.36, 16.16) to 24.24 (17.62, 36.4). This indicates that the prevalence rates of both men and women have increased, and the ASPR of women has always been higher than that of men (**Table 1**).

**Table 1:** The prevalence and mortality rates of thyroid cancer in China from 1990 to 2021.

Disease burden, per 100,000 no. (95%UI)	1990	2021
Prevalence case		
Both	0.87 (0.69,1.0)	3.88 (3.12,4.88)
Female	0.68 (0.49,0.84)	2.33 (1.69,3.46)
Male	0.19 (0.15,0.26)	1.55 (1.11,2.08)
ASPR		
Both	8.1 (6.41,9.66)	20.01 (16.14,25.23)
Female	13 (9.36,16.16)	24.24 (17.62,36.4)
Male	3.52 (2.77,4.73)	15.87 (11.35,20.96)
ASMR		
Both	0.47 (0.4,0.55)	0.39 (0.31,0.47)
Female	0.57 (0.43, 0.69)	0.32 (0.24,0.45)
Male	0.38 (0.31,0.51)	0.49 (0.33,0.64)

### 3.2 Mortality rate of thyroid in China from 1990 to 2021

In 1990 (**Figure 1B**), the crude mortality rate of the age group under 50 in China was close to 0. It began to change significantly at the age of 50. Before age 80, women had a higher mortality rate than men. After the age of 80, the mortality rate of men increased rapidly and reached its peak between the ages of 90 and 94. By 2021 (**Figure 1D**), the crude death rate for the under-50 age group remained close to zero. The mortality gap between men and women remained small until age 75. However, the mortality rate for men increased rapidly after the age of 75, reaching its peak at 90-94 years old. Furthermore, compared with 1990, the mortality rate of men is increasing while that of women is decreasing. Despite these changes, the overall mortality trends of both sexes remain similar.

The AMPR of the entire population decreased from 0.47 (0.4, 0.55) in 1990 to 0.39 (0.31, 0.47). The AMPR of men increased from 0.38 (0.31, 0.51) to 0.49 (0.33, 0.64), while that of women decreased from 0.57 (0.43, 0.69) to 0.32 (0.24, 0.45) (**Table 1**).

### 3.3 Analysis of risk factors for death from thyroid cancer

**Figure 2:**
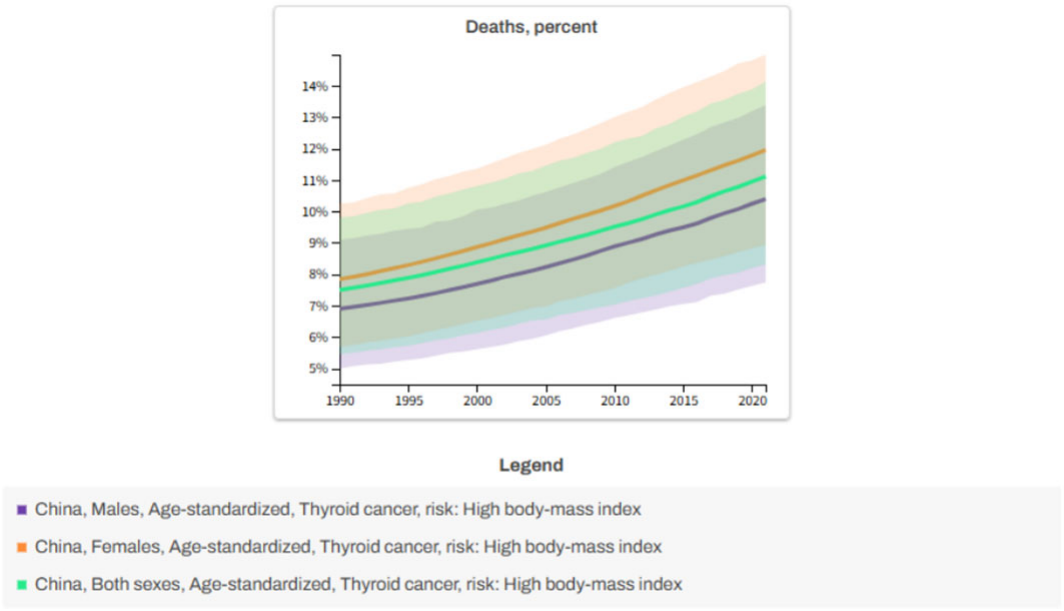
Analysis of risk factors for death. x-axis: Year; y-axis: Percentage of deaths.

Based on the data analysis of GBD 2021, the risk of death from thyroid cancer caused by high body mass index (BMI) in China showed a significant increasing trend from 1990 to 2021.As shown in **Figure 2**, the proportion of deaths from thyroid cancer due to high BMI in males increased from 6.89% to 10.40% (an increase of 50.97%), and in females it increased from 7.84% to 12% (an increase of 53.06%).

It is worth noting that metabolic risks associated with female obesity may be more sensitive to the prognosis of thyroid cancer. Overall, high BMI remains a key modifiable risk factor, with a more pronounced impact on women.

### 3.4 Relationship between BMI and thyroid cancer through MR analysis

Using BMI as the exposure variable and thyroid cancer as the outcome variable, select SNPs related to BMI as instrumental variables, and meet the following conditions: (1) Association hypothesis: Select SNP loci that are strongly correlated with exposure factors to ensure that the selected SNPs accurately reflect the genetic characteristics of thyroid cancer. (2) Independence hypothesis: SNP loci associated with confounding factors were excluded to eliminate the influence of external interference on the research results. (3) Exclusivity assumption: Only SNP loci that have an effect on the junction through exposure factors are retained to ensure the uniqueness of the causal relationship (32, 33).

When choosing tools, set the significance threshold at P<5×10-6 to preliminarily screen SNPS that are significantly related to BMI (34). Then, set the linkage disequilibrium parameter r2 = 0.001 and the genetic distance to 10,000 kb to avoid the bias caused by the linkage disequilibrium among SNPS. To conform to the exclusivity hypothesis, P<5×10-6 was set to eliminate SNPS related to the outcome (35). To meet the independence hypothesis, SNPS related to confounding factors need to be eliminated, and PheoScanner is used to query SNP-related models.

Through MR Analysis, there is a positive causal relationship between thyroid cancer and BMI(MR-Egger: OR=1.822, 95%CI: 0.1414-23.519, P=0.0646; IVW: OR=0.953, 95%CI: 0.358-2.2535, P=0.00242, Weighted mode: OR=0.403,95%CI: 0.048-3.395, P=0.0404), as shown in **Table 2**.

**Table 2:** Analysis of MR results with BMI as the exposure factor.

Id.exposure	Method	Nsnp	OR (95%CI)	Pval
BMI	MR-Egger	219	1.822 (0.1414-23.519)	0.0646
	IVW	219	0.953 (0.358-2.2535)	0.00242
	Weighted mode	219	0.403 (0.048-3.395)	0.0404

The IVW method is the most commonly used and statistically effective method in MR analysis. If the P value is less than 0.05, it is considered that there is statistical evidence supporting the causal effect of exposure on outcomes. However, the IVW method is highly sensitive to horizontal pleiotropy, and the estimation results of IVW may be biased. If the P-values of IVW and weighted median method are both significant and consistent in direction, this will enhance the conclusion of causal inference. The results showed that the P-values of IVW and Weighted mode were both less than 0.05, and the effect directions were consistent, indicating a causal relationship between BMI and thyroid cancer. This suggests that BMI may be an important factor in increasing the risk of thyroid cancer.

### 3.5 Prediction of multiple machine learning models based on SHAP interpretation

**Figure 3:**
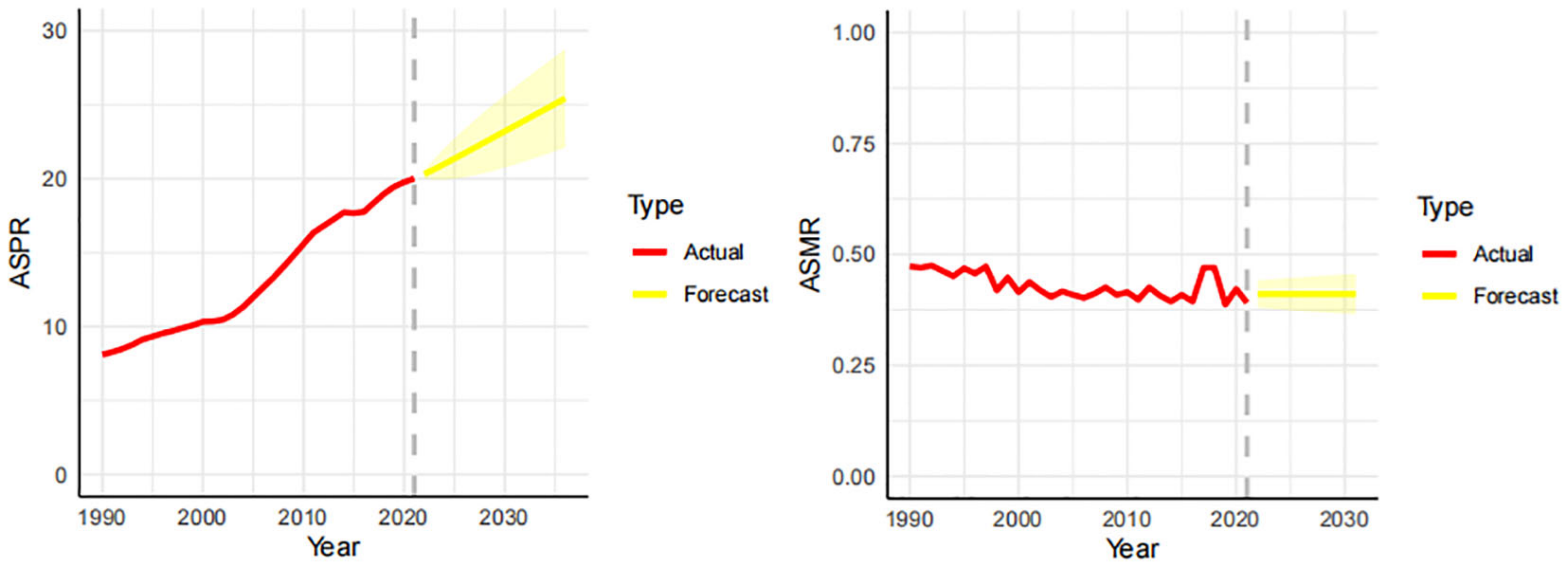
Forecast of the prevalence and mortality trends of thyroid cancer in China over the next 10 years (2021-2031). The real trends of prevalence and mortality in red lines. The yellow dotted lines and shaded areas represent the predicted trends and their 95%CI.

In order to better predict the future prevalence trend of thyroid cancer, we used ARIMA to predict the prevalence and mortality of thyroid cancer in the next 10 years. The prevalence is projected to rise from 20.01% in 2021 to 23.58% by 2031. The mortality rate is expected to remain stable, around 0.41%, during the same period. As shown in **Figure 3**.

Through the predictive analysis of GBD data, it was found that the prevalence of thyroid cancer still shows an increasing trend. To gain deeper insights into the current status and future progression of the disease, we built several machine learning models using hospital-based data. A total of 6,565 patients were included in this study, including 4,631 patients with malignant thyroid cancer and 1,934 patients with benign thyroid cancer. The data were randomly divided into the training set and the test set. The training set had a total of 5252 samples, and the test set had a total of 1313 samples.

For cases of missing data, Hot Deck Imputation was employed. Find the complete sample that is most similar to the missing value sample from the dataset, and use the value of this complete sample to fill in the missing value. The advantage of this method lies in its ability to preserve the original distribution of data, as it is filled based on actual observed values.

This study adopted a feature selection strategy that comprehensively considers clinical relevance, data availability and completeness, and model interpretability requirements when constructing a thyroid cancer benign and malignant prediction model. We carefully selected 9 clinical features closely related to thyroid disease diagnosis and patient basic information from hospital electronic medical records as model inputs.

Before conducting the model establishment analysis, the normality test of each index should be carried out first (36). When the P value of the normality test is greater than 0.05, it indicates that the index follows a normal distribution. The specific data are shown in **Table 3**. The indicators that follow a normal distribution include age, BMI, TG,FT3 etc.

**Table 3:** Comparison of indicators related to benign and malignant thyroid.

Eigenvalue	Total (N=6565)	Malignant (n=4631)	Benign (N=1934)	p.overall
sex, N (%):				0.727
male	1548 (23.580%)	1086 (23.451%)	462 (23.888%)	
female	5017 (76.420%)	3545 (76.549%)	1472 (76.112%)	
age, Median [Q1-Q3]	47.000 [38.000;56.000]	45.000 [36.000;54.000]	52.000 [43.000;60.000]	<0.001
BMI, N (%):				0.000
Overweight	4590 (69.916%)	4561 (98.488%)	29 (1.499%)	
normal	1960 (29.855%)	61 (1.317%)	1899 (98.190%)	
low weight	15 (0.228%)	9 (0.194%)	6 (0.310%)	
TG, Median [Q1-Q3]	19.250 [8.185;51.460]	15.530 [6.785;33.555]	44.200 [15.660;162.200]	<0.001
TP0AB, Median [Q1-Q3]	1.200 [0.500;11.918]	0.500 [0.500;7.660]	6.430 [0.500;16.780]	<0.001
TGAB, Median [Q1-Q3]	2.030 [0.500;16.053]	1.600 [0.500;14.175]	10.000 [1.110;18.778]	<0.001
TSH, Median [Q1-Q3]	1.672 [1.090;2.498]	1.749 [1.183;2.572]	1.466 [0.875;2.283]	<0.001
FT4, Median [Q1-Q3]	13.390 [12.180;15.000]	13.100 [12.010;14.395]	14.440 [12.718;16.600]	<0.001
FT3, Median [Q1-Q3]	4.365 [3.900;4.860]	4.410 [4.000;4.890]	4.200 [3.270;4.762]	<0.001

Six machine learning prediction models were constructed to predict the prevalence of thyroid cancer patients. During the model parameter tuning process, we utilized the grid search method. The aim is to find the optimal combination of hyperparameters through algorithms rather than manual trial and error, in order to achieve the best performance of the model. Predefine the discrete value range or list for each hyperparameter, and then iterate through all possible combinations of hyperparameters. For each combination, the model undergoes training and evaluation, followed by recording of performance metrics.

For the evaluation of the model, we utilized metrics such as AUC, accuracy, sensitivity, and specificity. The specific calculation formulas are as follows:


$$A\text{ccuracy}=\frac{TP+TN}{TP+TN+FP+FN}$$

$$Sensitivity=\frac{TP}{TP+FN}$$

$$Specificity=\frac{TN}{TN+FP}$$

True Positive (TP): The number of samples that are actually positive and also predicted as positive. True Negative (TN): The number of samples that are actually negative and also predicted as negative. False Positive (FP): The number of samples that are actually negative but are predicted as positive. False Negative (FN): The number of samples that are actually positive but are predicted as negative. **Table 4** shows the performance evaluation indicators of the six machine learning models constructed in this study (XGBoost, RandomForest, DecisionTree, GNB, MLP, SVM) in predicting the benign and malignant nature of thyroid cancer. The XGBoost model exhibits the best overall predictive performance. Its AUC value is as high as 0.999, accuracy is 0.984, sensitivity is 0.973, specificity is 0.988, positive predictive value is 0.968, negative predictive value is 0.990, and F1 score is 0.971. This indicates that XGBoost has extremely high accuracy and robustness in distinguishing benign and malignant thyroid cancer. In contrast, the performance of MLP and SVM is relatively poor. The AUC of MLP is only 0.661, the accuracy is 0.715, the sensitivity is only 0.469, and the F1 score is 0.480, indicating that its performance in this thyroid cancer benign and malignant prediction task is not ideal. Therefore, the XGBoost model based on gradient boosting demonstrated excellent performance in the benign and malignant prediction task of thyroid cancer in this study, far surpassing other traditional machine learning methods.

**Table 4:** Comparison of results from multiple models.

Models	AUC	Accuracy	Sensitivity	Specificity	Positive predictive value	Negative predictive value	F1 score
XGBoost	0.999	0.984	0.973	0.988	0.968	0.990	0.971
RandomForest	0.997	0.987	0.985	0.987	0.967	0.994	0.976
DecisionTree	0.979	0.983	0.970	0.988	0.970	0.988	0.970
GNB	0.991	0.984	0.989	0.982	0.953	0.996	0.971
MLP	0.661	0.715	0.469	0.811	0.492	0.796	0.480
SVM	0.838	0.769	0.831	0.743	0.569	0.917	0.675

**Figure 4:**
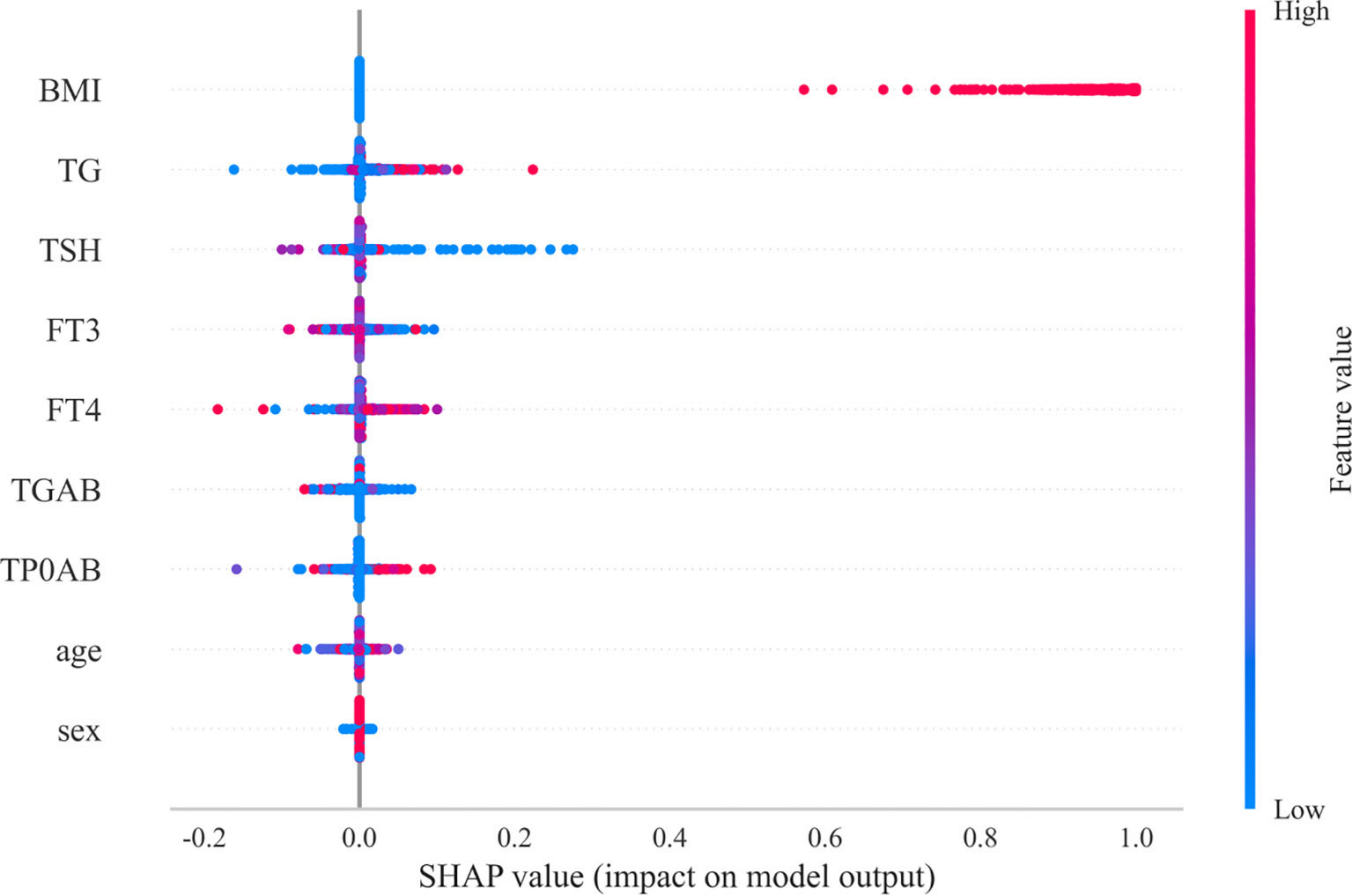
SHAP values.

The importance of features and the interpretability of the model are key aspects in evaluating performance. In the XGBoost model, BMI has the greatest influence among the nine common clinical features, as shown in **Figure 4**. The SHAP graph illustrates how each feature contributes to the model by displaying the distribution of SHAP values. The horizontal axis represents the SHAP value of the XGBoost model, and the color of the points indicates the size of the feature value. If the SHAP is positive, it indicates that the feature has a positive contribution.

From **Figure 4**, it can be clearly seen that the SHAP value distribution range of BMI is the widest, and its points are mainly concentrated in the larger positive areas. This indicates that BMI is the most important clinical feature affecting the prediction of benign and malignant thyroid cancer. A high BMI value is associated with a higher SHAP value, indicating that a high BMI significantly increases the likelihood of the model predicting malignancy. This is consistent with the conclusion drawn from Mendelian randomization analysis in the study that there is a positive causal relationship between BMI and thyroid cancer, further emphasizing the crucial role of BMI in the occurrence and development of thyroid cancer.

## 4 Discussion

This study aims to conduct an in-depth analysis of the disease patterns and future trends of thyroid cancer in China, and to construct an intelligent prediction model in combination with hospital electronic medical record data. It was found that the incidence of thyroid cancer in China has significantly increased, which is consistent with the global trend (37) and epidemiological analysis within China (38). The higher prevalence rate among women may be linked to hormone levels, autoimmune thyroid disease, and more frequent screenings. The peak age of onset for women has shifted from 55-59 years old in 1990 to 60-64 years old in 2021, which may reflect the aging of the population, the advancement of diagnostic techniques, and the evolution of the natural history of diseases. Although the prevalence rate continues to rise, the overall mortality rate remains relatively stable. This might be due to the popularization of early diagnosis, the advancement of treatment methods and the improvement of public health awareness. However, the rising trend of male mortality suggests that more in-depth attention should be paid to the risk factors and prognosis of thyroid cancer in men.

This study is the first to combine macroscopic GBD data with microscopic electronic medical record data from our hospital, providing a comprehensive perspective from the national level to clinical practice. This integration compensates for the limitations of a single data source (39), allowing research results to reflect the trend of disease burden nationwide and delve into the clinical characteristics of individual patients, providing a more solid foundation for developing precise prevention and control strategies.

This study introduced Mendelian randomization analysis to validate the relationship between BMI and thyroid cancer from a genetic causal perspective, which is relatively rare in previous research on thyroid cancer in China (40). The number of deaths from thyroid cancer caused by high BMI has surged in the past thirty years, which is consistent with the global trend of obesity and emphasizes the important role of obesity in the occurrence and development of thyroid cancer (41, 42). The results of MR analysis provide strong evidence that BMI is not only an accompanying phenomenon of thyroid cancer, but also an independent risk factor. This may be related to mechanisms such as chronic inflammation, insulin resistance, and endocrine disorders caused by obesity (43), providing new targets and stronger evidence support for the prevention and intervention of thyroid cancer.

This study constructed and compared various advanced machine learning models such as XGBoost and RandomForest (44), achieving high-precision prediction of benign and malignant thyroid cancer. The XGBoost model showed the best predictive performance, with an AUC value of up to 0.999, far exceeding the predictive ability of traditional statistical methods. More importantly, this study used interpretable methods such as SHAP values (45) to reveal key predictive features, clarifying that BMI is the most important clinical feature affecting thyroid cancer prediction, followed by thyroid function indicators such as TG, TSH, FT3, FT4, which are consistent with the physiological and pathological basis of thyroid disease (46). This interpretability not only enhances the transparency and clinical practicality of the model, but also provides a basis for clinical doctors to understand the decision-making process of the model, surpassing the limitations of traditional statistical methods and providing intelligent support for clinical decision-making.

This study analyzed the relationship between BMI and thyroid cancer by integrating GBD data and electronic medical record data and applying machine learning methods, successfully constructing a predictive model. Based on these findings, we propose the following multi-level implementation suggestions, aiming to translate research findings into practical actions to improve the prevention, diagnosis, and management of thyroid cancer. At the data level, we suggest further in-depth exploration of the GBD data, conducting refined regional and subgroup analyses to reveal the association patterns between BMI and thyroid cancer under different contexts. At the methodological level, we should actively promote the deployment and optimization of the machine learning prediction model developed in this study in clinical practice, integrate it into auxiliary diagnostic systems, and continuously collect new data for model retraining and interpretability research, ensuring its accuracy and practicality.

At the clinical practice level, it is recommended to focus on strengthening early screening and risk assessment for thyroid cancer, especially for individuals with a high BMI. This includes strengthening BMI monitoring during routine physical examinations and providing earlier or more frequent thyroid ultrasound examinations for overweight or obese individuals. At the policy and public health levels, it is recommended that the government and health departments formulate and refine national-level obesity prevention and control strategies, and integrate them organically with the prevention and control of thyroid diseases. Meanwhile, based on the macro data analysis of this study, it is advisable to guide the rational allocation of medical resources and allocate more resources to the screening and management of high-incidence areas or high-risk populations for thyroid cancer.

## 5 Conclusions

This study comprehensively analyzed the disease burden pattern of thyroid cancer in China from 1990 to 2021, revealing the significant upward trend of prevalence and the causal role of high BMI as an important controllable risk factor. By combining global disease burden data with hospital electronic medical records and applying advanced statistical and machine learning methods, we offered a comprehensive view of the epidemiological evolution of thyroid cancer in China. We also successfully built a high-precision prediction model for benign and malignant thyroid cancer. These findings provide a solid scientific basis for formulating more precise and effective strategies for the prevention, early diagnosis and management of thyroid cancer in China and even globally. This study emphasizes the importance of strengthening obesity intervention at the public health level and provides a feasible path for the use of artificial intelligence-assisted diagnosis in clinical practice. Looking to the future, the achievements of this study will lay a solid foundation for further in-depth exploration of the complex etiology of thyroid cancer, optimization of the diagnosis and treatment process, and ultimately improvement of patients’ health outcomes.

## Data Availability

The original contributions presented in the study are included in the article/**Supplementary Material**. Further inquiries can be directed to the corresponding author.
